# Simufilam Regulates Mitochondrial Function in HEK293T *TSC2* Knockout Cells

**DOI:** 10.3390/cells15201864

**Published:** 2026-10-14

**Authors:** Md. Monirul Islam, Aidan M. Sokolov, Lida Luo, Rolando Garcia-Milian, Katherine B Henke, Tukiet T. Lam, Angélique Bordey

**Affiliations:** 1 Departments of Neurosurgery and Cellular & Molecular Physiology, Wu Tsai Institute, Yale University School of Medicine, New Haven, CT 06520-8082, USA; 2 Bioinformatics Support Hub, Cushing/Whitney Medical Library, New Haven, CT 06520-8014, USA; rolando.milian@yale.edu; 3 Keck MS & Proteomics Resource, Yale School of Medicine, New Haven, CT 06511-6624, USAtukiet.lam@yale.edu (T.T.L.); 4 Department of Molecular Biophysics and Biochemistry, Yale School of Medicine, New Haven, CT 06520-8082, USA

**Keywords:** mTOR, TSC1, TSC2, tuberous sclerosis complex, simufilam, mitochondria

## Abstract

**Highlights:**

**What are the main findings?**

- Simufilam reduced elevated ATF4 expression without suppressing mTORC1 activity in *TSC2*-knockout HEK293T cells.
- Simufilam modified mitochondrial protein networks and restored mitochondrial membrane potential in *TSC2*-knockout cells.

**What are the implications of the main findings?**

- Simufilam may attenuate cellular stress and modify mitochondrial pathways in *TSC2*-deficient cells independently of direct mTORC1 inhibition.
- Targeting mitochondrial dysfunction may provide a complementary therapeutic strategy for TSC.

**Abstract:**

The small molecule simufilam, also called PTI-125, has been reported to reduce cell overgrowth and seizure activity in a mouse model of tuberous sclerosis complex (TSC). TSC is a multisystem developmental disorder caused by mutations in *TSC1* or *TSC2*, resulting in mTOR complex 1 (mTORC1) hyperactivity and downstream cellular changes that contribute to focal malformations of the developing cortex and abnormal growth throughout the body. Here, to better understand the role of simufilam at the cellular level, we investigated the impact of simufilam on the proteome of *TSC2* knockout (KO) HEK293T cells in vitro. We first validated that *TSC2* KO cells displayed increased mTORC1 activity and elevated expression of the stress-associated marker ATF4 compared with *TSC2* wild-type (WT) cells. Simufilam reduced ATF4 expression but did not attenuate mTORC1 activity. Next, using LC-MS/MS in *TSC2* KO cells, we identified 514 differentially abundant proteins following simufilam treatment. Pathway and protein–protein interaction analyses revealed coordinated changes in mitochondrial translation and bioenergetic pathways, including mitochondrial ribosomal proteins and respiratory-chain-associated proteins, as well as pathways related to preribosome biogenesis, rRNA processing, RNA metabolism, and to some extent RHO GTPase-actin signaling, and chromatin/transcriptional regulation. Selected proteins from these pathways, including MRPS30, MRPL43, MRPL16, ALDH1L2, NDUFA6, and RBM19, were further examined by immunoblotting and found to be altered by simufilam treatment. Functionally, simufilam restored mitochondrial membrane potential in *TSC2* KO cells. Overall, these findings support a potential mTORC1-independent role for simufilam in attenuating cellular stress and regulating mitochondrial function in TSC.

## 1. Introduction

Tuberous sclerosis complex (TSC) is a rare multisystem developmental disorder caused by pathogenic variants in *TSC1* or *TSC2*, which encode hamartin and tuberin respectively [1,2,3]. Loss of function of either gene disrupts the *TSC1/TSC2* complex and leads to canonical hyperactivation of the mechanistic target of rapamycin complex 1 (mTORC1), a central regulator of cell growth, cellular stress responses, metabolism, protein synthesis, and autophagy [1,2,4]. TSC is characterized by abnormal growth and lesion formation in multiple organs, including the brain, kidney, skin, heart, and lungs [3]. In the brain, malformations of cortical development, known as cortical tubers, are strongly associated with epilepsy and neurodevelopmental disability [5,6]. Although mTOR inhibitors have improved the treatment of some TSC manifestations, epilepsy and neurodevelopmental symptoms often remain incompletely controlled, supporting the need to identify therapeutic mechanisms beyond direct mTORC1 inhibition [7,8].

Although mTORC1 has been the major focus in TSC, additional pathways are dysregulated, including MEK/ERK signaling and downstream cytoskeletal regulatory proteins such as filamin A (FLNA) [9,10,11]. Pharmacological inhibition of MEK activity, knockdown of *FLNA*, or treatment with the small molecule simufilam, also known as PTI-125 and proposed to interact with FLNA, has been reported to reduce seizures in preclinical models of TSC and focal cortical dysplasia type II (FCDII) [9,12,13]. FCDII shares pathological features with cortical tubers and can result from pathogenic variants in mTOR pathway genes, including, but not limited to, *RHEB*, the canonical activator of mTORC1, as well as *TSC1* and *TSC2*, leading to mTORC1 hyperactivation [14]. Simufilam has also been reported to reduce neuronal soma enlargement and dendritic overgrowth [12]. However, the cellular pathways affected by simufilam in *TSC1*/*TSC2*-deficient cells remain poorly understood.

Here, we investigated the impact of simufilam on the proteome of *TSC2* knockout (KO) HEK293T cells. We first confirmed that *TSC2* KO cells exhibited increased mTORC1 activity, enlarged cell size, and elevated ATF4 expression compared with wild-type (WT) cells. mTORC1 has previously been linked to the activation of ATF4, a transcription factor involved in adaptive metabolic and stress-response programs, including amino acid metabolism, protein synthesis, and glutathione production [15]. We found that simufilam reduced ATF4 expression but did not significantly attenuate mTORC1 activity or cell size during 48 h of treatment. We then used LC-MS/MS-based proteomics to define simufilam-regulated protein networks in *TSC2* KO cells. Pathway and protein–protein interaction analyses identified coordinated changes in mitochondrial translation, respiratory-chain and oxidative phosphorylation pathways, nucleolar rRNA processing, and ribosome biogenesis, with additional effects on RHO GTPase-actin signaling and chromatin/transcriptional regulation. Finally, we validated selected proteins from these pathways and found that simufilam restored mitochondrial membrane potential in *TSC2* KO cells. Together, these data suggest that simufilam modifies stress-associated and mitochondrial programs in *TSC2*-deficient cells through mechanisms that appear to be independent of direct mTORC1 inhibition.

## 2. Methods

### 2.1. Cells and Cell Culturing

*TSC2* WT and *TSC2* KO HEK293T cells were obtained from the Tuberous Sclerosis Complex Alliance. These cells were cultured in Dulbecco’s Modified Eagle Medium (DMEM; Gibco, ThermoFisher Scientific, Grand Island, NY, USA), supplemented with 10% heat-inactivated fetal bovine serum (FBS; Gibco, ThermoFisher Scientific, Grand Island, NY, USA) and 1% penicillin–streptomycin (Gibco, ThermoFisher Scientific, Grand Island, NY, USA). Cells were maintained at 37 °C in a humidified incubator with 5% CO_2_. For routine maintenance, cells were seeded into T75 culture flasks and passaged upon reaching approximately 80–90% confluency. For subculturing, cells were washed once with calcium- and magnesium-free Dulbecco’s Phosphate-Buffered Saline (DPBS; Gibco, ThermoFisher Scientific, Grand Island, NY, USA), incubated with 0.05% Trypsin-EDTA solution (Gibco, ThermoFisher Scientific, Grand Island, NY, USA) until detached, and subsequently neutralized with complete growth medium prior to reseeding. *TSC2* KO and WT HEK293T cells were seeded at a density of 1.05 × 10^6^ cells per 10 cm culture plate in Dulbecco’s Modified Eagle Medium (DMEM) supplemented with 5% FBS and 1% penicillin–streptomycin.

### 2.2. Simufilam (PTI-125) Drug Reconstitution and Treatment

Simufilam was purchased from MedChemExpress company (Monmouth Junction, NJ, USA) and was reconstituted in dimethyl sulfoxide (DMSO) to prepare a 100 mM stock solution. This stock was diluted to 10 mM in saline and used within one week. The vehicle solution was prepared using an equivalent dilution of DMSO in saline. All reported simufilam concentrations refer to the salt form, and the final DMSO concentration in the culture medium was 0.01% in both treatment groups. The simufilam stock solution was stored at 4 °C, protected from light, and prepared fresh each week. The final simufilam working solution was prepared daily from the stock solution. Immediately following seeding, cells were treated with either 10 µM simufilam or DMSO as vehicle control. After 24 h, fresh media and simufilam were exchanged. Cells were harvested 48 h after seeding and lysates were prepared for downstream experiments.

### 2.3. Immunoblotting

Cells were first detached using 0.05% trypsin-EDTA and washed twice with cold phosphate-buffered saline (PBS) to ensure complete removal of residual trypsin and media. After washing, the cells were lysed using RIPA lysis buffer (Thermo Scientific, Rockford, IL, USA), supplemented with the protease and phosphatase inhibitor cocktails (Thermo Scientific, Rockford, IL, USA). The total amount of protein was measured by the Pierce BCA protein assay kit. Equal amounts of protein were loaded onto an SDS–PAGE gel for separation. After electrophoresis, the gel was rinsed for 2–3 min in water to remove any residual running buffer. Proteins were then transferred to a PVDF membrane at 100 V for approximately 120 min. Upon completion of the transfer, the membrane was rinsed again for 2–3 min in water to remove traces of transfer buffer. Next, the membrane was blocked in a 5% non-fat milk (Bio-Rad, Hercules, CA, USA) in TBS (150 mM NaCl, 25 mM Tris-HCl, pH 7.5). After another brief rinse, the membrane was incubated overnight at 4 °C with primary antibodies listed in Supplemental Table S1. Subsequently, the membrane was incubated with anti-rabbit and anti-mouse conjugated with horseradish peroxidase as the secondary antibodies. As part of our immunoblotting workflow, proteins were initially transferred onto a single membrane, which was then cut into separate sections to allow probing with different primary antibodies, including the antibody used to detect the loading control. Blots were visualized using the Clarity Western ECL Substrate (Bio-Rad, Hercules, CA, USA) and acquired via a Chemiluminescence Imaging System (G: Box mini, Cambridge, UK).

### 2.4. Proteomics

Samples: HEK293T *TSC2* null cells (5 × 10^5^ cells/pellet; eight total pellets; four treated with DMSO and four treated with simufilam) were provided in 10 µL of 1× PBS containing protease and phosphatase inhibitors. Samples were stored at −80 °C prior to processing.

Sample preparation: RIPA buffer (300 µL) (Thermo Scientific, cat. #89901) containing 1× HALT protease/phosphatase inhibitor cocktail (Thermo Scientific, cat. #78447) was added to each sample. The samples were sonicated at 10 amps for two rounds of 15 s with 1 s on/off pulses, storing the samples on ice between rounds. The samples were then centrifuged at 4 °C for 10 min at 20,500 *g*. A 175 µL aliquot of supernatant was removed from each sample, and proteins were precipitated using chloroform/methanol/water. Protein pellets were resuspended in 52 µL of 8 M urea/400 mM ammonium bicarbonate and protein concentrations were measured via absorbance at 280 nm using a Nanodrop spectrophotometer (Thermo Fisher Scientific, Wilmington, DE, USA). A 150 µg aliquot of protein was removed from each sample and reduced with 4 mM dithiothreitol (Thermo Scientific, Rockford, IL, USA, cat. #20290) for 30 min at 37 °C. Samples were cooled then alkylated with 8 mM iodoacetamide (Sigma-Aldrich, St. Louis, MO, USA, cat. #I1149) for 30 min at room temperature in the dark. Proteins were digested at 37 °C via overnight incubation with 3 µg endoproteinase LysC (New England Biolabs, Ipswich, MA, USA, cat. #P8109S) followed by a 6.5 h incubation with 3 µg trypsin (Promega, Madison, WI, USA, cat. #V5113). Trifluoroacetic acid (TFA) was added to each sample to a final concentration of 1% and samples were desalted using BioPureSPN Macro columns (The Nest Group, Inc., Ipswich, MA, USA, cat. #HMM-S18V). Desalted peptides were dried via vacuum centrifugation and resuspended in 70 mM L-glutamic acid/65% acetonitrile (ACN)/2% TFA in water (loading buffer) prior to phosphopeptide enrichment using TopTip TiO_2_ columns (Glygen, Columbia, MD, USA, cat. #TT1TIO.96). Briefly, columns were conditioned with loading buffer prior to applying peptide samples and centrifuging. Samples were reloaded to maximize phosphopeptide yield and flowthrough was saved for LC-MS/MS analysis. TiO_2_ columns were washed three times with loading buffer followed by three times with 65% ACN/2% TFA in water prior to eluting phosphopeptides with three aliquots of 2% NH_4_OH in water. Enriched and flowthrough fractions were dried via SpeedVac and resuspended in 2% ACN/0.2% TFA in water. Peptide concentrations in the flowthrough fractions were measured via absorbance at 280 nm. Peptide amounts in the enriched fractions were estimated as 4% of the amount in the corresponding flowthrough sample. Peptide dilutions (0.06 µg/µL) were prepared for LC-MS/MS analysis and Pierce retention time calibration mixture (Thermo Scientific, Rockford, IL, USA, cat. #88321) (12.5 fmol/µ) was added to each sample prior to injection.

LC-MS/MS data acquisition: LC-MS/MS data were acquired on a Thermo Scientific Q Exactive HF-X mass spectrometer coupled to a Waters M-Class UPLC system (Waters corporation, Milford, MA, USA). Peptides (300 ng) were loaded onto a nanoEase M/Z Symmetry C18 trap column (100 Å, 5 µm, 180 μm × 20 mm) (Waters, cat. #186008821) at a flow rate of 5 µL/min, with 0.5% B for 3 min, and separated using a peptide BEH C18 analytical column (130 Å, 1.7 μm, 75 μm × 250 mm) (Waters, cat. #186008795) (35 °C). The compositions of mobile phases A and B were 0.1% formic acid in water and 0.1% formic acid in ACN, respectively. The peptides were eluted at a flow rate of 300 nL/min with a gradient starting at 3% B, increasing to 6% B over 2 min, then to 25% B over 173 min and 40% B over 20 min. The gradient was then ramped to 90% B over 5 min and held for 10 min before the column was equilibrated to starting conditions. Precursor MS1 scans (profile) were collected from 350 to 1500 *m*/*z* at a resolution of 120,000. The AGC target was set to 3 × 10^6^ and the maximum injection time was 50 ms. Data-dependent MS2 scans (top 20; centroid) were collected from 200 to 2000 *m*/*z* at a resolution of 30,000. Precursors with 2 ≤ *z* ≤ 7 were selected for HCD fragmentation with a collision energy setting of 30% and an isolation window of 1.4 *m*/*z*. The AGC target was set to 1 × 10^5^ and the maximum injection time was 50 ms. Dynamic exclusion was enabled with an exclusion duration of 15 s. One blank injection was performed between enriched and flowthrough fractions of the same sample and four blank injections were performed between injections of different samples.

Proteomics data analysis: Label-free quantification (LFQ) was performed using Progenesis QI software (Nonlinear Dynamics, Newcastle upon Tyne, UK, version 4.2) as described in [16]. For each condition, data from the phosphopeptide-enriched and the flowthrough fractions were analyzed separately, and the quantitative results were combined prior to export. Briefly, Progenesis QI performed chromatographic retention time alignment between runs prior to data filtering and peak picking. Peak picking was performed using default sensitivity settings and features with retention times from 10 to 200 min and with charge states less than eight were retained. An abundance normalization factor was calculated for each run to account for variability in peptide loading on the LC-MS system. MS/MS spectra with rank less than 11 were then exported as an MGF file for searching via the Mascot search engine (Matrix Science, London, UK, version 2.8). Data were searched against the SwissProt *Homo sapiens* database (May, 2023) and a database containing the Pierce retention time calibration peptides. Trypsin was selected as the enzyme, up to two missed cleavages were allowed, and precursor and product ion mass tolerances were set to 10 ppm and 0.02 Da, respectively. Carbamidomethylation of cysteine was set as a fixed modification and oxidation of methionine, acetylation of lysine and protein N-termini, deamidation of asparagine and glutamine, and phosphorylation of serine, threonine, and tyrosine were set as variable modifications. The PSM-level FDR was set to 2%. Search results were exported from Mascot as XML files and imported into Progenesis QI for feature identification. Relative quantification was performed using non-conflicting peptides and quantified proteins/peptides were exported into Microsoft Excel for downstream analysis. Only proteins with at least two unique peptides across the enriched/flowthrough samples were considered for subsequent analysis.

### 2.5. Bioinformatic Analysis

Differential abundance and pathway analysis: Proteomics differential abundance was calculated by Student’s *t*-test on log2-transformed values. Differentially abundant proteins (DAP) were considered those with absolute fold-change ≥ 1.5 and Benjamini–Hochberg False Discovery Rate (FDR) *p* < 0.05 between the experimental groups. Qlucore Omics Explorer version 3.9 (Qlucore, Lund, Sweden) was used for visualization including Principal component analysis, hierarchical clustering heatmap, and the global proteomics change across the groups by a volcano plot.

For functional analysis, DAP was uploaded to the manually curated knowledgebase Ingenuity Pathway Analysis (IPA) (Qiagen, Redwood City, CA, USA, Build 9.0, 2020–2024). The Core Analysis function within IPA was used for overrepresentation analysis. Enriched pathways were those with an overlap *p*-value < 0.05 (FDR *p* < 0.05) of the right-tailed Fisher’s exact test.

Gene Set Enrichment Analysis (GSEA) desktop (Broad Institute, Cambridge, MA, USA and University of California San Diego, La Jolla, CA, USA, version 4.4.0, build 18) was used to run the GSEA pre-ranked analysis [17]. Proteins were ranked using a signed metric calculated as

ranking score = log_2_ fold-change × [−log_10_(FDR-adjusted *p*-value)]

The log_2_ fold-change was calculated for simufilam-treated cells relative to DMSO-treated *TSC2* KO cells. Positive scores therefore indicated increased protein abundance following simufilam treatment, whereas negative scores indicated decreased abundance. The ranked list was evaluated against the following gene sets: h.all.v2023.2, c2.all.v2023.2, c5.all.v2023.2, and c7.immunesigdb.v2023.2 with the following parameters: nperm 1000, set_min 10, set_max 500, random seed: 1715716977453.

Interaction network and cluster analysis: Protein–protein interaction network and cluster analysis was performed on differentially abundant proteins by STRING database (version 12.0) [18]. Interaction network was generated with a minimum required interaction score confidence of 0.4 (medium). Clustering analysis was done on STRING using the unsupervised Markov Clustering Algorithm (inflation parameter 3) [19]. Global interaction network and clusters were exported to and visualized on the Cytoscape (version 3.10.5), an open-source platform for complex network analysis and visualization [20]. String protein–protein cluster analysis can be assessed at https://version-12-0.string-db.org/cgi/network?networkId=b1CwQZVhhb3C (accessed on 10 January 2025).

### 2.6. Mitochondrial Membrane Potential Analysis

*TSC2* WT and *TSC2* KO HEK293T cells were seeded onto 6 cm culture plates at a density of 3.92 × 10^5^ cells per plate in DMEM supplemented with 5% FBS and 1% penicillin–streptomycin. Immediately after seeding, cells were treated with either 10 µM simufilam or DMSO as vehicle control. After 24 h of incubation, the culture medium and simufilam treatment were refreshed. After a total treatment period of 48 h, cells were washed with PBS, and mitochondrial membrane potential was assessed using the TMRM Assay Kit (Abcam, Cambridge, UK, cat. #ab228569). Cells were stained with 50 nM tetramethylrhodamine methyl ester (TMRM), together with 3 µM Calcein AM (ThermoFisher Scientific, cat. #C1430) and 1 µg/mL Hoechst 33342 (Thermo Scientific, cat. #62249), for 20 min at 37 °C. The staining solution was then removed and replaced with the live-cell imaging buffer supplied with the TMRM assay kit. Cells were imaged using a Leica TCS SP8 confocal laser scanning microscope (Leica Microsystems, Wetzlar, Germany). TMRM and Hoechst 33342 fluorescence intensities were quantified on a per-cell basis in Fiji (ImageJ, version 1.54t) with Calcein fluorescence used to identify individual, live cells. TMRM intensity was then normalized to Hoechst 33342 intensity. For analysis, TMRM intensity was measured in 3 plates (biological replicates) per group. Five to six fields of images per plate were taken and nine cells per field were drawn and measured. As a total, 17 (6 × 2 + 5) fields per group were analyzed and there were 45 or 54 cells per plate, resulting in 153 cells per group.

### 2.7. Statistical Analysis (Except for Proteomic Analysis)

The graphs of experimental results were generated using GraphPad 10 Prism. A *p*-value < 0.05 was considered statistically significant. Data normality was determined using the D’Agostino and Pearson test and the Shapiro–Wilk test. Statistical analyses were also performed in GraphPad 10 Prism using Welch’s *t*-test or one-way ANOVA.

## 3. Results

### 3.1. Simufilam Reduces Elevated ATF4 Expression Without Attenuating mTORC1 Hyperactivity in TSC2 KO Cells

**Figure 1:**
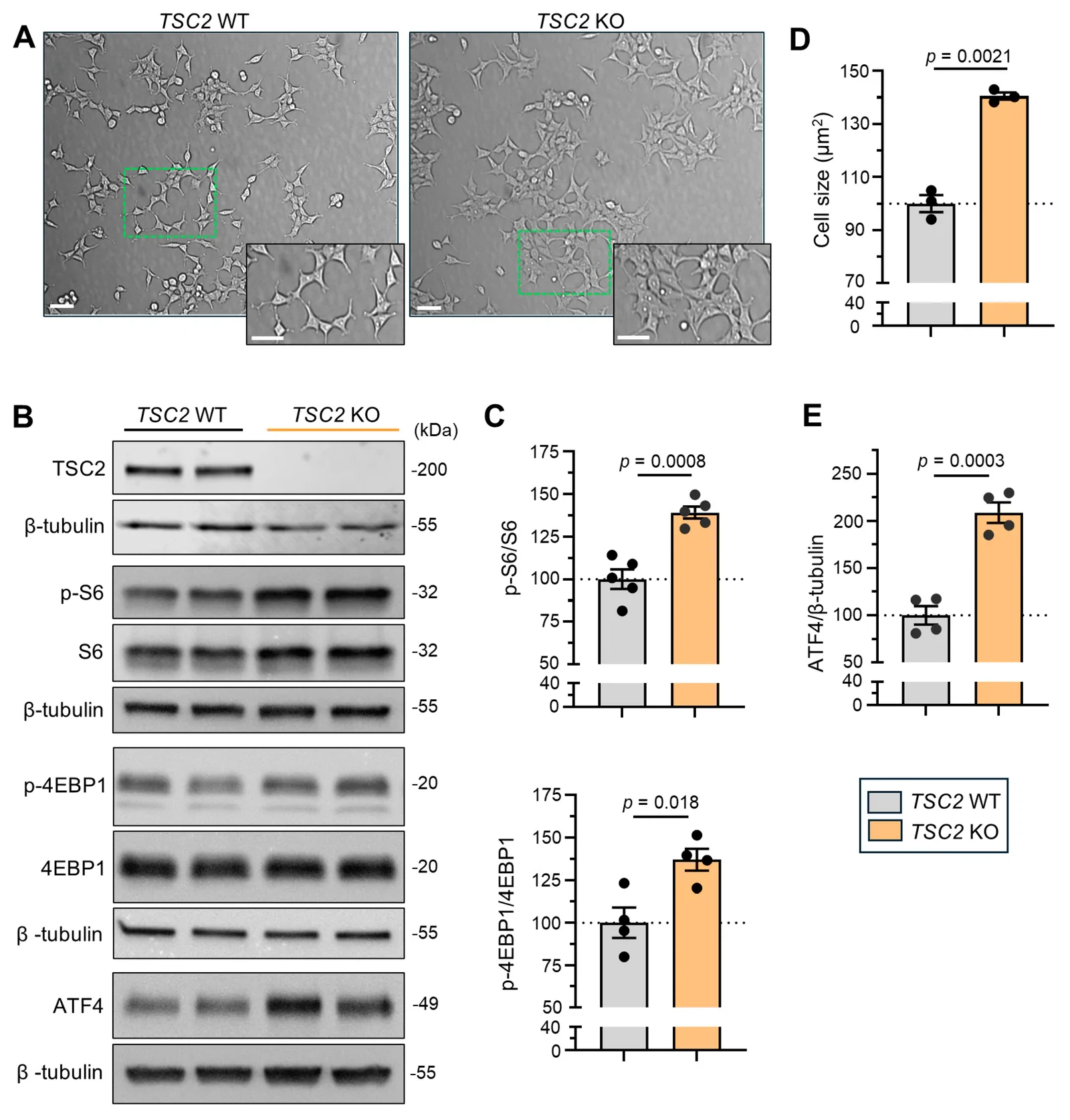
*TSC2* KO HEK293T cells display increased mTORC1 activity, ATF4 expression, and cell soma size. (**A**) Representative images of *TSC2* wild-type (WT) cells (Left) and *TSC2* KO cells (Right). Inset shows higher magnification images. Scale bars: 55 and 25 (inset) µm. (**B**) Representative Western blot images of proteins and phospho-proteins listed on the left. (**C**) Quantification of p-S6/S6 ratio and p-4E-BP1/4E-BP1 ratio. (**D**) Quantification of cell soma size. (**E**) Quantification of ATF-4 levels. Data are presented as mean ± SEM. N = 4–5 per condition as shown on the plots. Statistical analysis: unpaired *t*-test with Welch’s correction. *p*-values are listed on top of each plot. The original blots for all the biological replicates are provided in Supplementary Figure S1.

**Figure 2:**
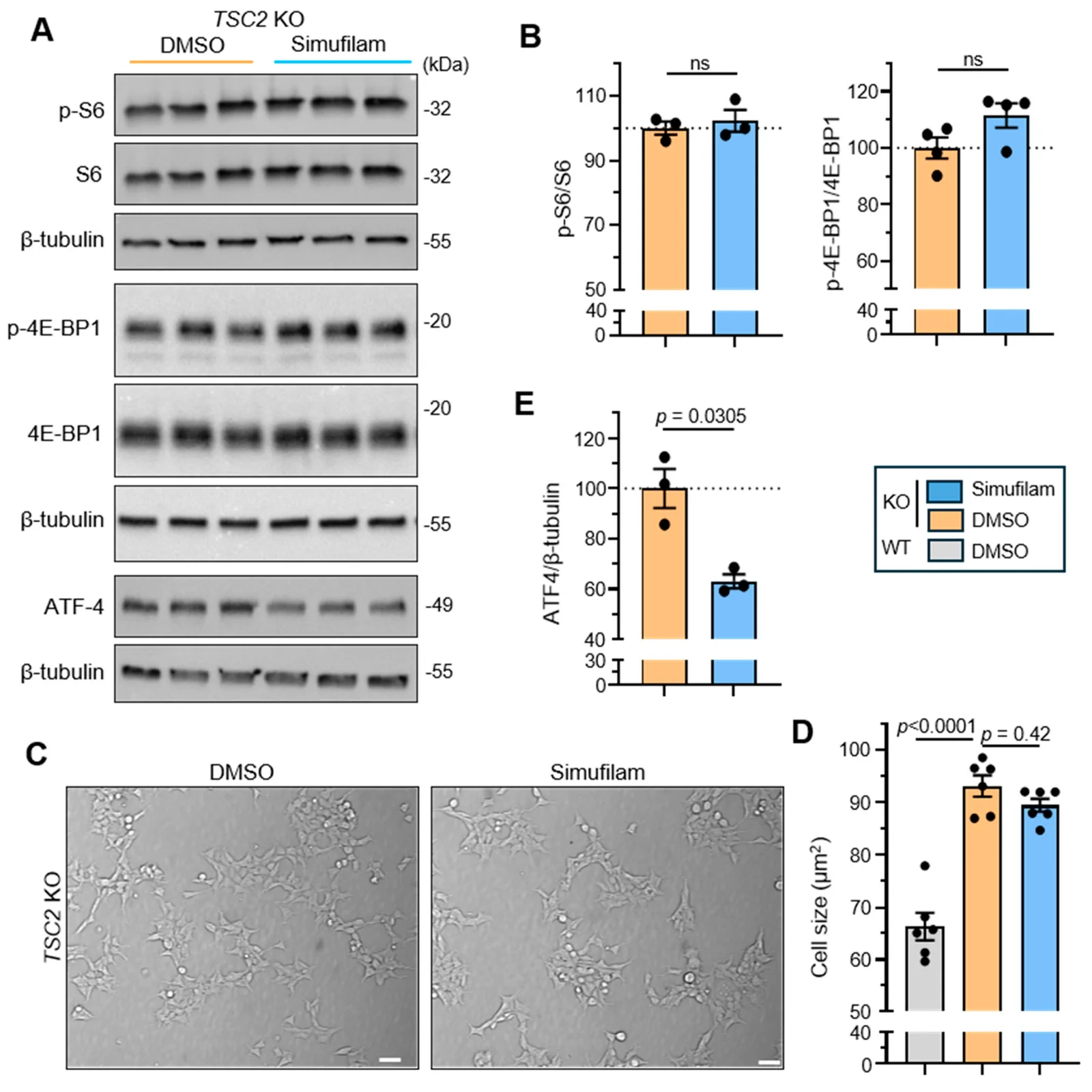
Simufilam reduces elevated ATF4 expression without attenuating mTORC1 hyperactivity in *TSC2* KO cells. (**A**) Representative Western blot images of proteins and phospho-proteins listed on the left. (**B**) Quantification of p-S6/S6 ratio (Left) and p-4E-BP1/4E-BP1 ratio (Right). (**C**) Representative images of *TSC2* KO cells treated with DMSO or Simufilam. Scale bar: 40 µm. (**D**) Quantification of cell soma size (Right). (**E**) Quantification of ATF4 levels. Data are presented as mean ± SEM. N = 4–5 per condition as shown on the plots. Statistical analysis: unpaired *t*-test with Welch’s correction (**B**,**E**) and one-way ANOVA with Tukey post hoc giving F (2,15) = 50.25, *p* < 0.0001 (**D**). *p*-values are listed on top of each plot. The original blots for all the biological replicates are provided in Supplementary Figure S2.

*TSC2* KO HEK293T cells were generated by Dr. Nellist [21] and are available through the TSC Alliance Biosample Repository. The loss of *TSC2* was routinely examined using immunoblotting for *TSC2* as illustrated in Figure 1B. To validate mTORC1 hyperactivity in *TSC2* KO cells relative to wild-type (WT) cells, we first optimized the culture conditions. mTORC1 activity was assessed by immunoblotting for phosphorylated ribosomal protein S6 (p-S6) and total S6, as well as phosphorylated 4E-BP1 and total 4E-BP1, followed by quantification of the phospho/total ratios. Because cells reached confluency by 48 h, a condition known to suppress mTORC1 signaling through cell–cell contact inhibition [22,23], we plated cells at a lower density of 1.05 × 10^6^ cells per 10 cm culture plate. FBS was also reduced to 5% to minimize basal mTORC1 activation in WT cells. Under these conditions, cells were approximately 50 to 60% confluent at the time of collection (Figure 1A). In addition, pS6/S6 and p4E-BP1/4E-BP1 ratios were consistently increased 48 h after plating (Figure 1B,C statistical values in the figures), indicating increased mTORC1 activation. Therefore, this culture condition was used for the remaining studies. Consistent with increased mTORC1 activity, the soma of *TSC2* KO cells was significantly larger than that of *TSC2* WT cells (Figure 1A,D and Figure S1). Finally, immunoblotting for ATF4, a transcription factor induced by ER stress and activated downstream of mTORC1 [15], showed that ATF4 protein abundance was increased by approximately 150% in *TSC2* KO cells compared with WT cells (Figure 1E). Having established conditions where *TSC2* KO cells exhibit the expected mTORC1 activation, we assessed whether 48 h simufilam treatment altered key molecular or cellular features of these cells. Cells were treated with 10 µM simufilam for 2 days, and mTORC1 activity readouts and ATF4 expression were examined (Figure 2A). Simufilam did not significantly alter the p-S6/S6 or p-4E-BP1/4E-BP1 ratios (Figure 2B), nor did it significantly reduce cell soma size (Figure 2C,D). However, simufilam significantly reduced ATF4 expression by approximately 37% (Figure 2E), suggesting that simufilam attenuates a stress-adaptive transcriptional program in *TSC2* KO cells. The original blots for all the biological replicates are provided in Supplementary Figure S2.

### 3.2. Proteomics Analysis of Simufilam Treatment Identified 514 Differential Expressed Proteins Associated with Strong Enrichment in Mitochondrial Programs and RNA Processing

**Figure 3:**
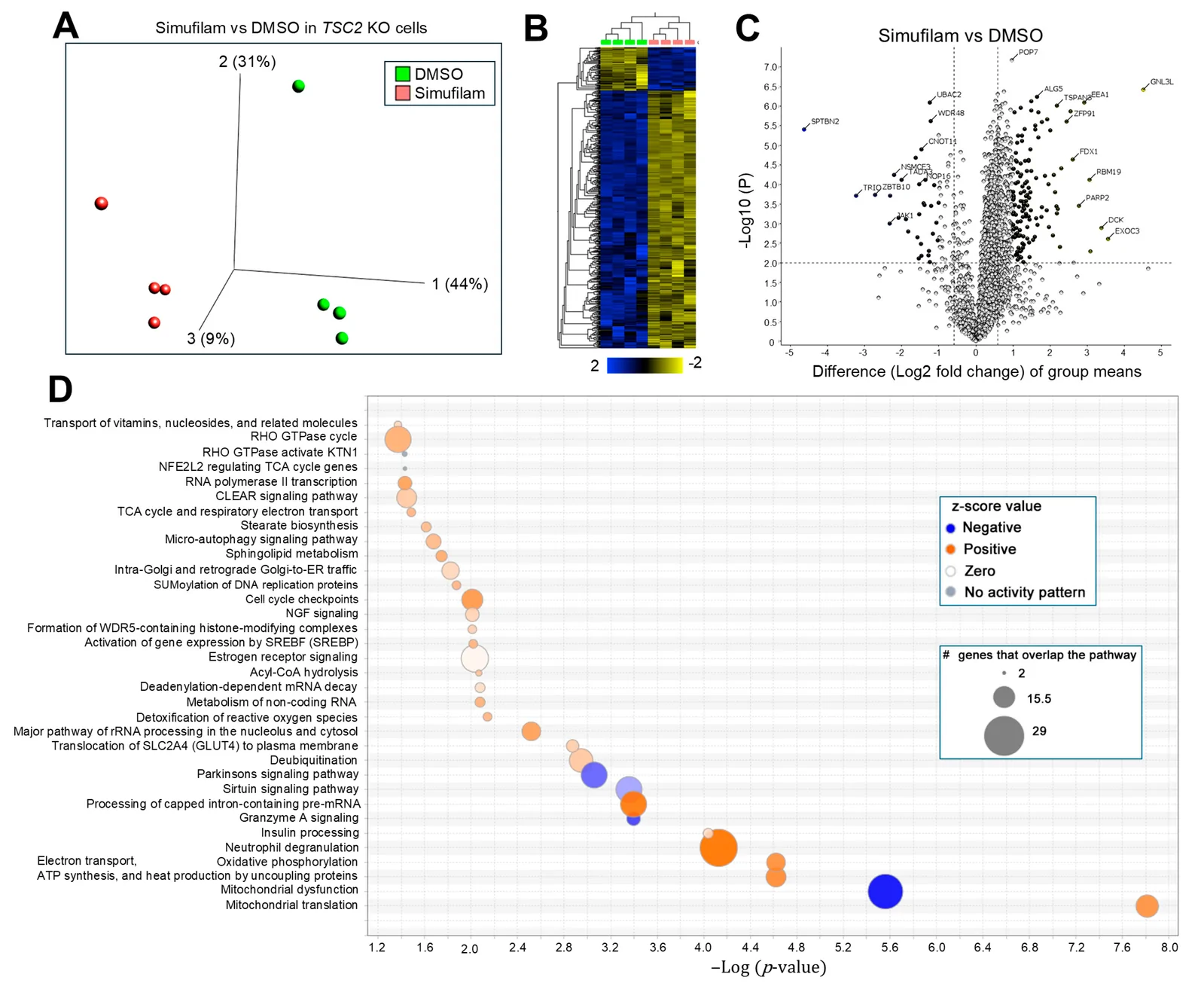
Proteomic profiling reveals Simufilam-induced protein expression changes and pathway modulation in *TSC2* KO cells. (**A**) PCA of global proteomic profiles from DMSO- and simufilam-treated *TSC2* KO cells. (**B**) Heatmap of differentially expressed proteins. Differentially expressed proteins were identified using a two-tailed unpaired *t*-test with correction for FDR, applying an FDR threshold of <0.05 and a fold-change cutoff of ≥1.5. (**C**) Volcano plot showing differential protein expression between DMSO- and simufilam-treated *TSC2* KO cells. (**D**) Pathway enrichment analysis of differentially expressed proteins. Enriched pathways are ranked by statistical significance based on −log10(*p*-value).

To identify the impact of simufilam on the proteome of *TSC2* KO cells, we performed LC-MS/MS in *TSC2* KO cells treated with vehicle (DMSO) or simufilam (10 µM) for 48 h (Supplementary Table S2, n = 4 replicates per condition). The principal component analysis (PCA) of the proteomic dataset revealed distinct clustering of samples according to treatment conditions. Vehicle/untreated samples and simufilam-treated samples separated in PCA space, indicating that 48 h simufilam treatment altered the global proteomic profile of *TSC2* KO cells (Figure 3A). We identified 514 differentially abundant proteins illustrated on a heatmap (two-tailed unpaired Student’s *t*-test FDR *p* < 0.05, absolute fold-change ≥ 1.5, Figure 3B) and volcano plot (Figure 3C). The fold-change of the significantly altered proteins is provided in Supplementary Table S3. Ingenuity Pathway Analysis (IPA) of the simufilam versus DMSO comparison identified 34 significantly enriched canonical pathways, ranked by enrichment significance as −log10 (FDR *p*-value) (Figure 3D and Figure S4). The most significantly enriched pathways were strongly dominated by mitochondrial biology, with the top-ranked pathways including mitochondrial translation (FDR *p* = 1.54 × 10^−8^), mitochondrial dysfunction (FDR *p* = 2.75 × 10^−6^), electron transport, ATP synthesis, and heat production by uncoupling proteins (FDR *p* = 2.39 × 10^−5^), and oxidative phosphorylation (FDR *p* = 2.39 × 10^−5^). These pathways were associated predominantly with positive activation z-score values (orange bubbles), suggesting predicted activation or increased activity of mitochondrial translational and bioenergetic programs following simufilam treatment. Bubble size reflects the number of genes overlapping each pathway, with larger bubbles representing broader gene coverage; thus, enrichment of several mitochondrial pathways was supported not only by statistical significance but also by multiple overlapping genes. Additional significantly enriched pathways implicated RNA metabolism and protein synthesis, including the processing of capped intron-containing pre-mRNA (FDR *p* = 3.98 × 10^−4^), major pathway of rRNA processing in the nucleolus and cytosol (FDR *p* = 3.02 × 10^−3^), metabolism of non-coding RNA (FDR *p* = 8.31 × 10^−3^), deadenylation-dependent mRNA decay (FDR *p* = 8.31 × 10^−3^), and RNA polymerase II transcription (FDR *p* = 3.71 × 10^−3^), suggesting coordinated effects on RNA maturation, ribosome biogenesis, transcript turnover, and transcriptional regulation. Pathways related to sirtuin signaling (FDR *p* = 4.36 × 10^−4^), detoxification of reactive oxygen species (FDR *p* = 7.24 × 10^−4^), TCA cycle and respiratory electron transport (FDR *p* = 3.23 × 10^−2^), and NFE2L2-regulated TCA cycle genes (FDR *p* = 3.71 × 10^−2^) further support the effect of simufilam on mitochondrial metabolic and redox-associated processes. Lower-ranked but significant pathways, including RHO GTPase activate KTN1 (FDR *p* =3.71 × 10^−2^), RHO GTPase cycle (FDR *p* = 4.26 × 10^−2^), Golgi-to-ER traffic (FDR *p* = 1.51 × 10^−2^), CLEAR signaling (FDR *p* = 3.54 × 10^−2^), and micro-autophagy signaling (FDR *p* = 2.08 × 10^−2^), suggest additional effects on cytoskeletal regulation, intracellular trafficking, and cellular quality-control pathways. Overall, the pathway profile indicates that simufilam treatment is associated primarily with the activation of mitochondrial and bioenergetic programs, accompanied by broader changes in RNA processing, ribosome-related pathways, and redox regulation.

As an independent gene set-based approach, we performed GSEA and considered gene sets significant at an FDR q-value < 0.25. The GSEA results closely mirrored the IPA canonical pathway analysis, with the strongest positively enriched signatures centered on mitochondrial localization and bioenergetic function, including GOCC_mitochondrial_matrix (Normalized Enrichment Score NES = 3.86, FDR q = 0), GOCC_mitochondrial_protein (NES = 3.75, FDR q = 0), WONG_mitochondria_gene_module (NES = 3.42, FDR q = 1.81 × 10^−4^), ALLMARK_oxidative phosphorylation (NES = 3.20, FDR q = 6.0 × 10^−5^), REACTOME_the TCA cycle_and_respiratory electron transport (NES = 3.18, FDR q = 1.04 × 10^−4^), and WP_electron transport chain_oxphos system in mitochondria (NES = 2.61, FDR q = 7.83 × 10^−3^) (Supplementary Table S5). GSEA also supported the IPA and STRING (see below) findings related to translation and ribosome-associated programs, with, for example, enrichment of GOCC_ribosomal subunit (NES = 3.28, FDR q = 9.0 × 10^−5^). Examples of a corresponding enrichment plot, heatmap, and mitochondrial bubble plots showed coordinated increases in respiratory-chain, oxidative phosphorylation, and mitochondrial-associated proteins in the simufilam group compared with DMSO, rather than isolated changes in individual proteins (Supplementary Figure S3). Together, these GSEA results provide orthogonal support for the IPA findings and strengthen the conclusion that simufilam treatment is associated with the activation of mitochondrial translation and bioenergetic pathways, accompanied by broader effects on RNA/ribosome-related and cellular stress-associated programs in *TSC2* KO cells.

**Figure 4:**
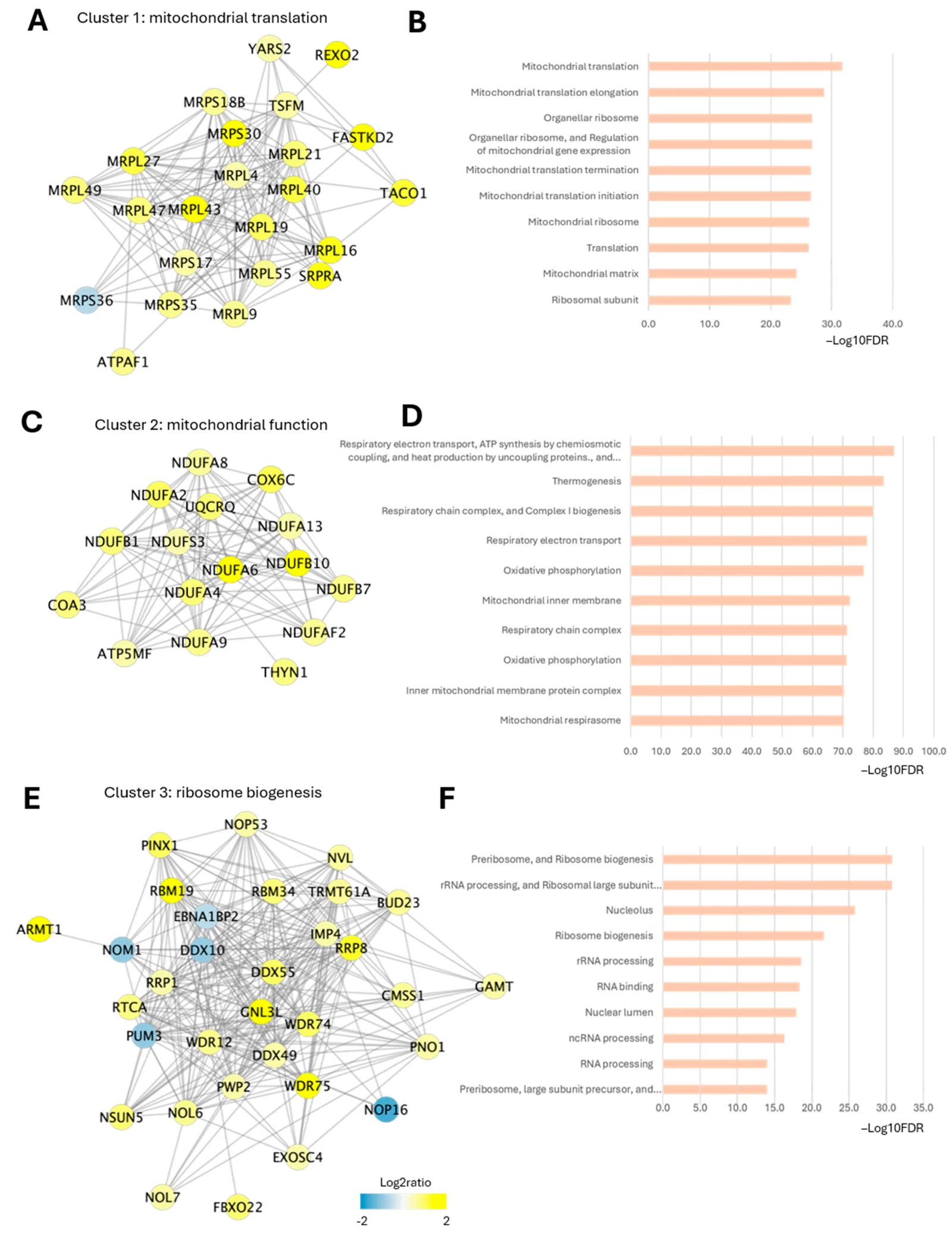
Proteomics analysis of simufilam treatment identifies differential protein clusters enriched in mitochondrial pathways and RNA processing. (**A**,**C**,**E**) STRING protein–protein interaction networks of differentially expressed proteins, showing three highly enriched clusters. Node colors indicate log_2_ ratio fold-change. (**B**,**D**,**F**) Top enriched GO, Reactome, and STRING pathways for each cluster. Enrichment significance was determined using false discovery rate (FDR)-adjusted *p*-values.

To further refine the pathway-level findings, we performed STRING protein–protein interaction network analysis, which identified three highly significant and biologically coherent protein clusters (Figure 4, Supplementary Table S6). Cluster 1 included 22 of 514 identified proteins (4.3%), with strong enrichment for mitochondrial translation and mitochondrial ribosomal proteins (Figure 4A), and was the most statistically significant module; the top GO biological process was mitochondrial translation with 18 proteins and an FDR of 8.32 × 10^−32^, and Reactome pathways for mitochondrial translation elongation, initiation, and termination were also highly significant (Figure 4B, only 10 enriched pathways are illustrated). A second cluster (cluster 2) included 16 of 514 proteins (3.1%) and represented a complementary mitochondrial module enriched for respiratory electron transport, ATP synthesis by chemiosmotic coupling, oxidative phosphorylation, mitochondrial inner membrane, respiratory-chain complex, and complex I biogenesis (Figure 4C), with the top STRING enrichment for respiratory electron transport/ATP synthesis reaching an FDR of 6.59 × 10^−27^ (Figure 4D). Together, these two clusters accounted for 38 of 514 proteins (7.4%) and closely matched the dominant IPA signature related to mitochondrial translation, mitochondrial function, oxidative phosphorylation, and respiratory electron transport. A third cluster (cluster 3) included 32 of 514 proteins (6.2%) and was enriched for nucleolar ribosome biogenesis (Figure 4E) with an enrichment FDR of 2.28 × 10^−22^, preribosome assembly, rRNA processing with an FDR of 2.79 × 10^−19^, ncRNA processing, and RNA processing; STRING terms for rRNA processing/ribosomal large subunit biogenesis and preribosome/ribosome biogenesis reached FDR values of 1.63 × 10^−31^ (Figure 4F). These results support the pathway-level enrichment of rRNA processing and ribosome-related processes.

Two additional clusters identified secondary but biologically meaningful modules (Supplementary Table S6). Cluster 4 included 17 of 514 proteins (3.3%) and was enriched for RHO GTPase signaling, including the RHO GTPase cycle, RHO/ROCK signaling, small GTPase-mediated signal transduction, and actin cytoskeleton organization (Supplementary Figure S4A,B). The top Reactome term, RHO GTPase cycle, included 10 proteins and reached an FDR of 1.63 × 10^−9^, indicating that this cluster represents a coherent cytoskeletal/signaling module corresponding to the lower-ranked RHO GTPase-related pathways observed in the IPA. Cluster 5 included 17 proteins (3.3%) and was enriched for chromatin and transcriptional regulatory processes, including histone modification and methylation, heterochromatin assembly, chromatin organization, and RNA polymerase II-dependent transcriptional regulation (Supplementary Figure S4C,D); the top GO term, histone modification, included nine proteins and reached an FDR of 1.97 × 10^−7^.

Collectively, STRING network analysis resolved the proteomic changes into highly significant coordinated modules centered on mitochondrial translation, oxidative phosphorylation/respiratory-chain function and nucleolar rRNA processing/ribosome biogenesis, with more minor modules centered on RHO GTPase-actin cytoskeletal signaling, and chromatin/transcriptional regulation.

### 3.3. Simufilam Restores Mitochondrial Membrane Potential in TSC2 KO Cells

**Figure 5:**
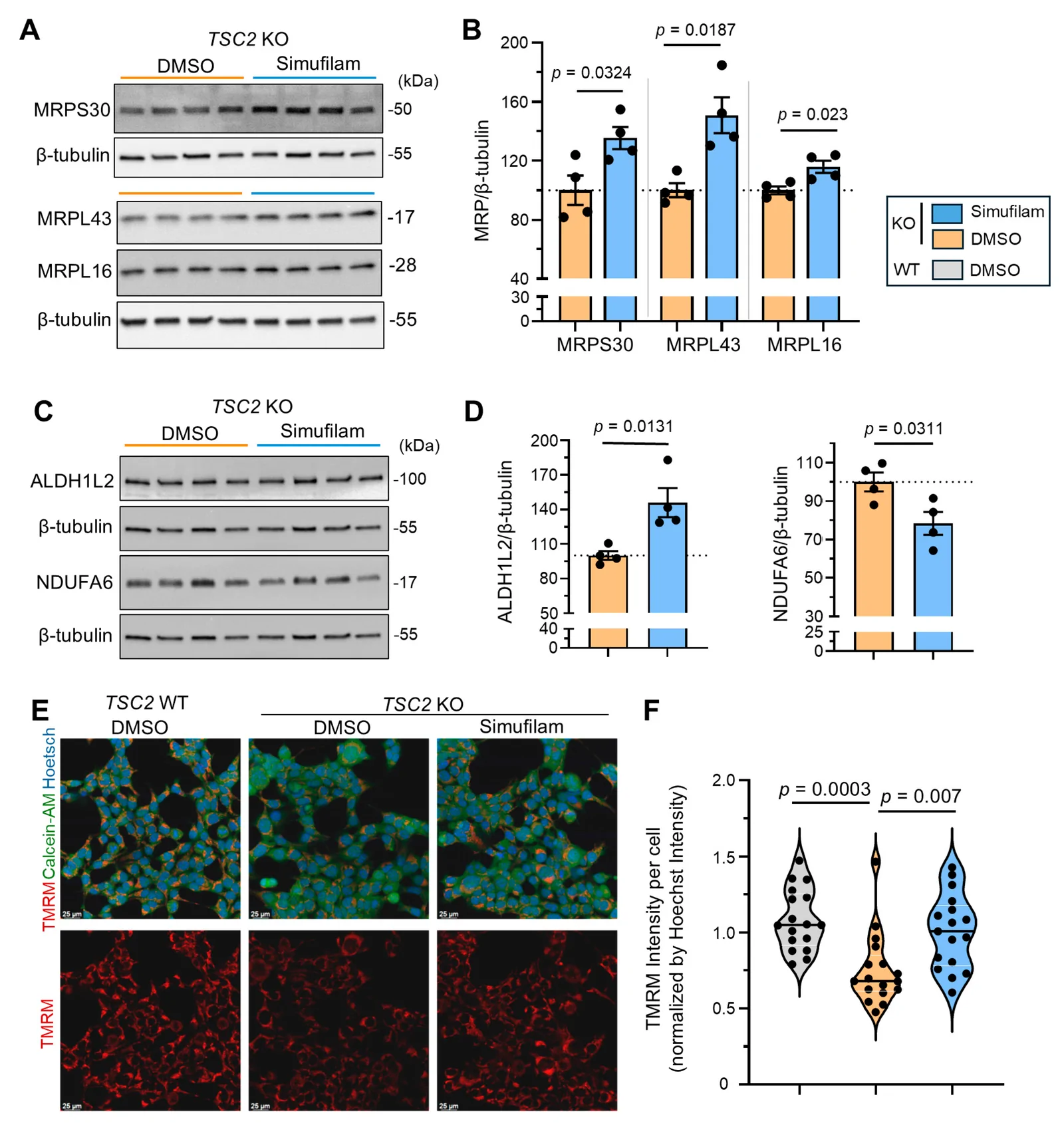
Simufilam alters mitochondrial protein expression and restores mitochondrial membrane potential in *TSC2* KO cells. (**A**) Immunoblot images of MRPS30, MRPL43, and MRPL16 in *TSC2* KO cells treated with DMSO or simufilam. (**B**) Quantification of the blots. (**C**,**D**) Immunoblot images of ALDH1L2 and NDUFA6 (**C**) and quantification (**D**). (**E**) Representative images of TMRM (Red), Calcein (green) and Hoechst (Blue) in *TSC2* WT and *TSC2* KO cells treated with DMSO or simufilam. (**F**) Quantification of TMRM intensity. Data are presented as mean ± SEM; N = 4 per condition for (**A**–**D**). Statistical significance was assessed by unpaired *t*-test with Welch’s correction (**B**,**D**) and one-way ANOVA with Bonferroni, F (2,48) = 9.7, *p* = 0.0003 (**F**). The original blots are provided in Supplementary Figures S5 and S6.

We focus on validating proteins that belong to the mitochondrial translation and function clusters illustrated in Figure 4. Using immunoblotting, we found that simufilam treatment significantly increased the expression of three mitochondrial ribosomal proteins, MRPS30 (1.35-fold), MRPL43 (1.51-fold) and MRPL16 (1.16-fold) (Figure 5A,B), that displayed a significant fold-increase in the proteomic dataset (see Supplementary Table S3). When comparing *TSC2* KO versus WT, the levels of these proteins were significantly decreased in *TSC2* KO (see Supplementary Figure S5). We examined the expression of the mitochondrial one-carbon/folate metabolism enzyme, aldehyde dehydrogenase 1 family member L2, ALDH1L2), and the accessory subunit of Complex I, NDUFA6. Both were increased by simufilam treatment in the proteomic dataset. Although ALDH1L2 was not listed as part of the cluster 2 (mitochondrial function), it can impact mitochondrial function through mitochondrial one-carbon metabolism and redox control and was highly increased in the proteomic dataset. Using immunoblotting, we validated that simufilam significantly increased the expression of ALDH1L2 (1.46-fold) but decreased NDUFA6 expression by 0.78-fold (Figure 5C,D). The levels of these two proteins were similar in *TSC2* KO and WT cells (Supplementary Figure S6). We finally validated a couple of proteins in the third cluster (ribosome biogenesis), including the nucleolar GTPase involved in ribosomal pre-rRNA processing, GNL3L and the nucleolar RNA binding proteins RBM19 that displayed large fold-increase in the proteomic dataset. RMB19, but not GNL3L, was significantly increased by simufilam (Supplementary Figure S7).

Considering the impact of simufilam on the expression of mitochondrial proteins, we examined whether simufilam could alter mitochondrial membrane potential. For this, we measured TMRM, a cell-permeant fluorescent dye that accumulates in active mitochondria as a readout of mitochondrial membrane potential (Figure 5E). We found that *TSC2* KO cells displayed a decreased TMRM signal that was fully restored with simufilam treatment (Figure 5F), indicating recovery of mitochondrial membrane potential.

## 4. Discussion

In the culturing condition, *TSC2* KO HEK293T cells displayed increased mTORC1 activity as well as ATF4 and cell soma size compared to WT cells. In *TSC2* KO HEK293T cells, simufilam reduced the stress-associated transcription factor ATF4 but did not significantly attenuate canonical mTORC1 activity, as assessed by p-S6/S6 and p-4E-BP1/4E-BP1, nor did it reduce cell size over the 48 h treatment period. These findings suggest that simufilam does not primarily act through acute suppression of mTORC1 signaling in this model but may instead modulate downstream or parallel stress-adaptive pathways that are altered in *TSC2*-deficient cells.

Proteomic analysis further supported this interpretation by revealing coordinated changes in protein networks related to mitochondrial translation, respiratory-chain and oxidative phosphorylation pathways, nucleolar rRNA processing, and ribosome biogenesis. STRING network analysis showed that these changes were organized into highly connected functional modules rather than isolated protein changes, strengthening the conclusion that simufilam affects coherent biological programs. The enrichment of mitochondrial ribosomal proteins and respiratory-chain-associated proteins was particularly notable, given the close relationship between mTORC1 signaling, cellular metabolism, redox control, and mitochondrial physiology. The biological relevance of these proteomic changes was supported by immunoblot validation of selected mitochondrial and ribosome-associated proteins. In addition, simufilam restored mitochondrial membrane potential in *TSC2* KO cells. Together with the reduction in ATF4 expression, these data suggest that simufilam attenuates cellular stress, modifies mitochondrial protein networks, and may improve certain aspects of mitochondrial function in *TSC2*-deficient cells without normalizing mTORC1 hyperactivity. This distinction is important because many TSC manifestations, particularly neurological symptoms, remain incompletely controlled by mTOR inhibitors, highlighting the need to identify mechanisms that act outside canonical mTORC1 suppression.

Mitochondria are therapeutic targets across a broad range of neurological, cardiovascular, metabolic, and malignant disorders, but the desired direction of mitochondrial modulation is disease- and cell-context dependent [24,25,26,27,28]. In vulnerable nonmalignant cells, therapeutic strategies may aim to preserve or restore mitochondrial homeostasis, while, in cancer cells, mitochondrial metabolism and respiration can support tumor growth and may therefore represent therapeutic targets for inhibition [24,25,26]. For example, supinoxin suppressed small-cell lung cancer growth by inhibiting DDX5-dependent mitochondrial respiration [24]. Conversely, mitochondrial proteins and mitochondrial-targeted compounds are being investigated as therapeutic targets in neurodegenerative, metabolic, cardiovascular, and inflammatory disorders [25,27,28]. Thus, mitochondrial modulation should not be considered universally beneficial or detrimental. In the present transformed, non-cancer-derived *TSC2*-deficient cell model, simufilam modified mitochondrial protein networks and restored mitochondrial membrane potential, although these results do not establish a general improvement in mitochondrial function.

Although the proteomic and functional data identify simufilam-regulated pathways in *TSC2*-deficient cells, they do not establish FLNA as the direct molecular target of simufilam. Demonstrating FLNA engagement would require additional studies, such as direct binding or target-engagement assays, and FLNA loss- and rescue-of-function experiments, that are outside the scope of this study. Nevertheless, several simufilam-regulated pathways identified here overlap with known FLNA biology. FLNA has been reported to localize to the nucleolus and regulate the expression of ribosomal proteins as well as RNA polymerase I-dependent rRNA transcription, a central component of ribosome biogenesis [29,30]. FLNA has also been implicated in mitochondrial dynamics through its hypoxia-induced interaction with Drp1, which promotes mitochondrial fission-associated cellular dysfunction [31]. In addition, FLNA has been shown to regulate RhoA activation in neural progenitors and neutrophils, linking FLNA to RHO GTPase-dependent cytoskeletal signaling [32,33]. Thus, while the present study did not examine if simufilam acts through FLNA, it identified biological pathways that are consistent with known FLNA-regulated processes and support further mechanistic studies to determine whether FLNA contributes to simufilam’s effects in *TSC2*-deficient cells.

Several limitations should be considered. The study was performed in HEK293T cells, which provide a tractable model of TSC2 loss but do not fully recapitulate the cellular context of cortical tubers or TSC-associated neuronal dysfunction. Therefore, findings in the KO condition may not fully reflect those in neurons. ATF4 was not detected by LC-MS/MS, likely because its abundance was below the detection limit of the untargeted proteomics workflow. In agreement with this, ATF4 was detectable by Western blot only after long exposure, supporting its relatively low abundance despite induction in *TSC2* KO cells. This also suggests that our LC-MS/MS analysis may have missed the effects of simufilam on low-abundance proteins. Further, the mechanism(s) by which simufilam regulates ATF4 and identifies protein networks remains unresolved, including whether these effects involve FLNA-dependent pathways or other cellular targets. Future studies should be aimed at measuring phosphorylated eIF2α and relevant upstream kinases to clarify the mechanism underlying the reduction in ATF4. An additional limitation relates to the interpretation of the mitochondrial membrane potential measurements. Although simufilam restored the reduced TMRM signal in *TSC2* KO cells, membrane potential represents only one aspect of mitochondrial physiology and does not directly measure respiratory-chain complex activity, oxygen consumption, ATP production, or respiratory supercomplex assembly. In addition, simufilam reduced the expression of NDUFA6, an accessory subunit required for normal complex I catalytic function, despite restoring membrane potential. This apparent divergence cannot be explained by the present experiments because immunoblotting does not determine NDUFA6 incorporation into assembled complex I or measure complex I enzymatic activity. The present findings also do not identify whether compensatory changes in complexes II, III, or IV contribute to the restoration of membrane potential. Future studies should thus assess respiratory-chain complex activities, oxygen-consumption rates, cellular ATP production, and respiratory supercomplex assembly. These experiments are beyond the scope of the current study and may be more informative in disease-relevant models, because HEK293T cells are metabolically adaptable and do not recapitulate the neuronal context of TSC. Finally, the use of a single simufilam concentration and treatment duration limits our ability to determine the concentration and temporal dependence of the observed effects or to exclude concentration-specific responses. Future studies should examine multiple concentrations and time points in disease-relevant cellular models. Collectively, we plan to investigate stress-response and mitochondrial pathways in additional models, including iPSC-derived neurons from individuals with TSC.

## 5. Conclusions

Overall, the reported findings support a potential role for simufilam in modifying stress-associated and mitochondrial programs in *TSC2*-deficient cells through mechanisms that appear distinct from direct mTORC1 inhibition.

## Data Availability

The proteomics dataset has been submitted to ProteomeXchange via the PRIDE database. To access the data, log in to the PRIDE website using the following details: Project accession: PXD083450 and Token: jH0iHKU1inBC.

## Supplementary Materials

The following supporting information can be downloaded at: https://www.mdpi.com/article/10.3390/cells15201864/s1.
