# Supplementary material for: Simufilam Regulates Mitochondrial Function in HEK293T TSC2 Knockout Cells
Source: Cells. 2026 Oct 14;15(20):1864. doi: 10.3390/cells15201864 (PMC13702739; doi:10.3390/cells15201864)
Supplement: Supplementary file 1 [file cells-15-01864-s001.zip › Supplementary Information.pdf]

Supplementary Information

Simufilam regulates mitochondrial function in tuberous sclerosis complex condition

Md. Monirul Islam [1,†], Aidan M. Sokolov [1,†], Lida Luo [1], Rolando Garcia-Milian [2], Katherine B Henke [3], Tukiet T. Lam [3,4] and Angélique Bordey [1,*]

List of supplementary documents:

Supplementary Figures S1-S7
Supplementary Table S1
Supplementary Tables S2-S6 are provided as individual Excel files.

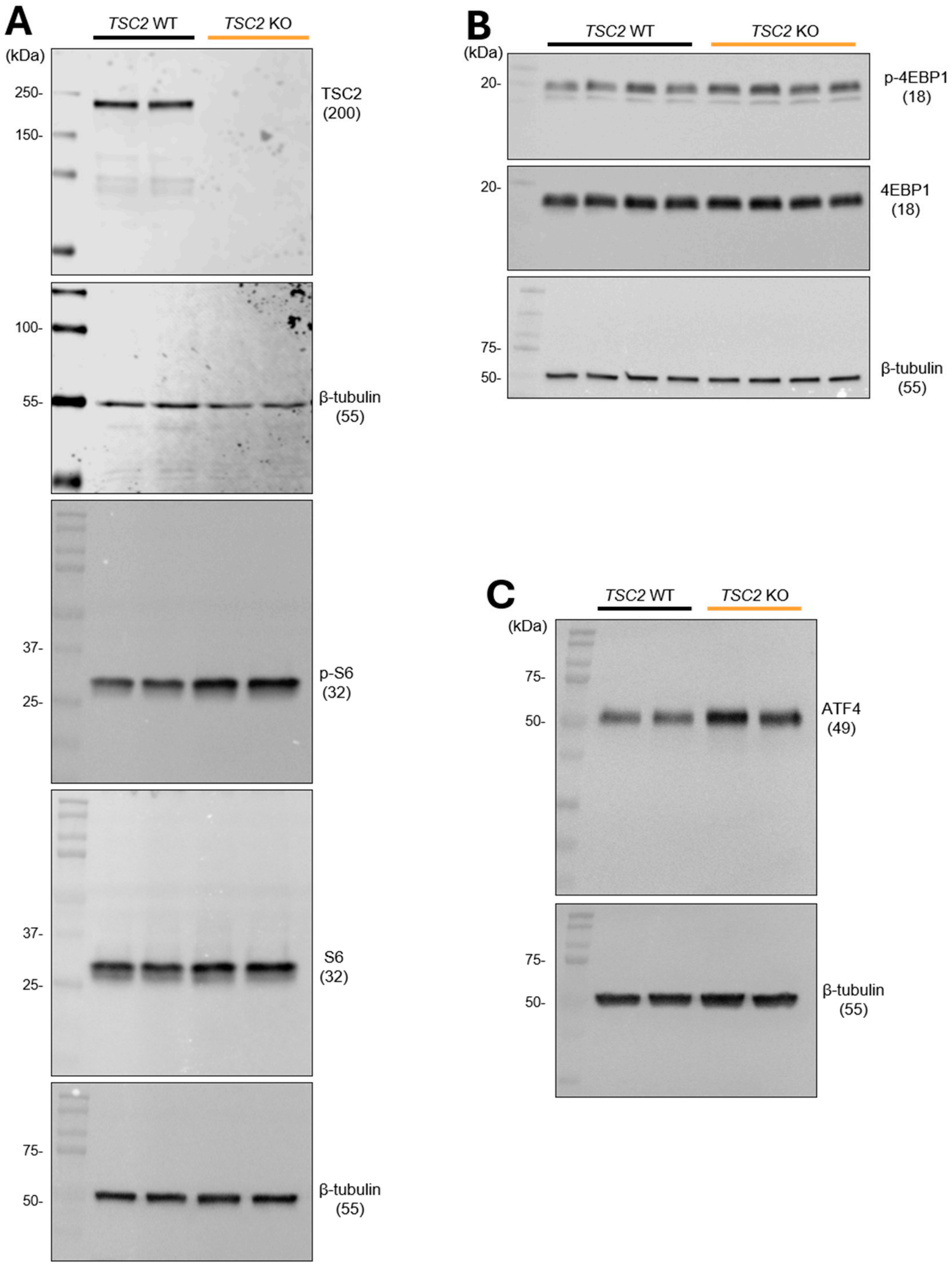

Supplementary Figure S1: *TSC2* KO HEK293T cells display increased mTORC1 activity and ATF4 expression. Representative western blot images of **(A)** TSC2 and P-S6/S6, **(B)** P-4EBP1/4EBP1, and **(C)** ATF4 in *TSC2* wild-type (WT) and *TSC2* KO cells.

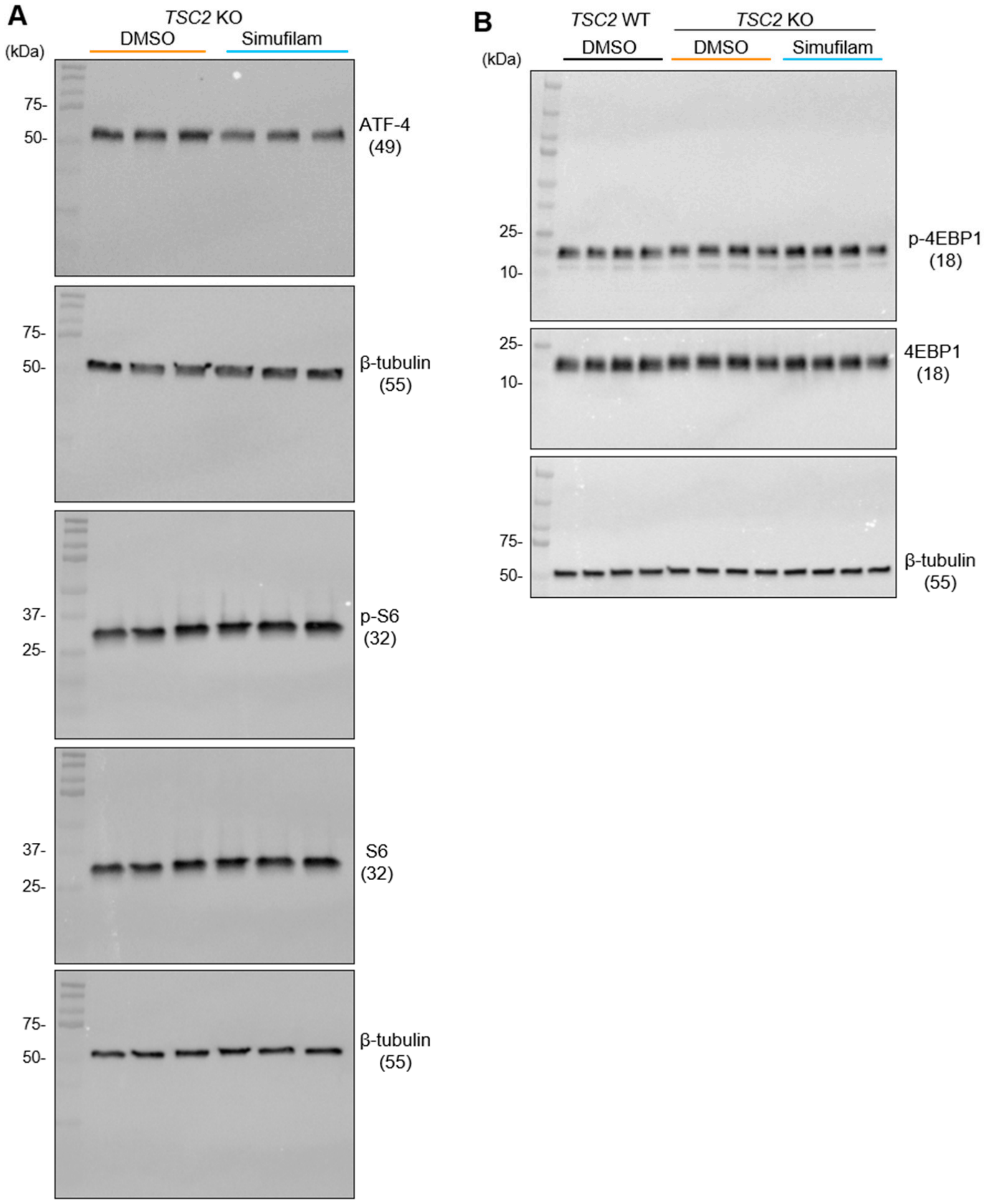

Supplementary Figure S2: Simufilam reduces elevated ATF-4 expression without attenuating mTORC1 hyperactivity in *TSC2* KO cells. (A and B) Representative western blot images of ATF-4, p-S6 and S6 (A), and p-4E-BP1 and 4E-BP1 (B) with their respective loading control β-tubulin (on the same blot) in *TSC2* KO cells treated with DMSO or simufilam. Each protein group was on different blots.

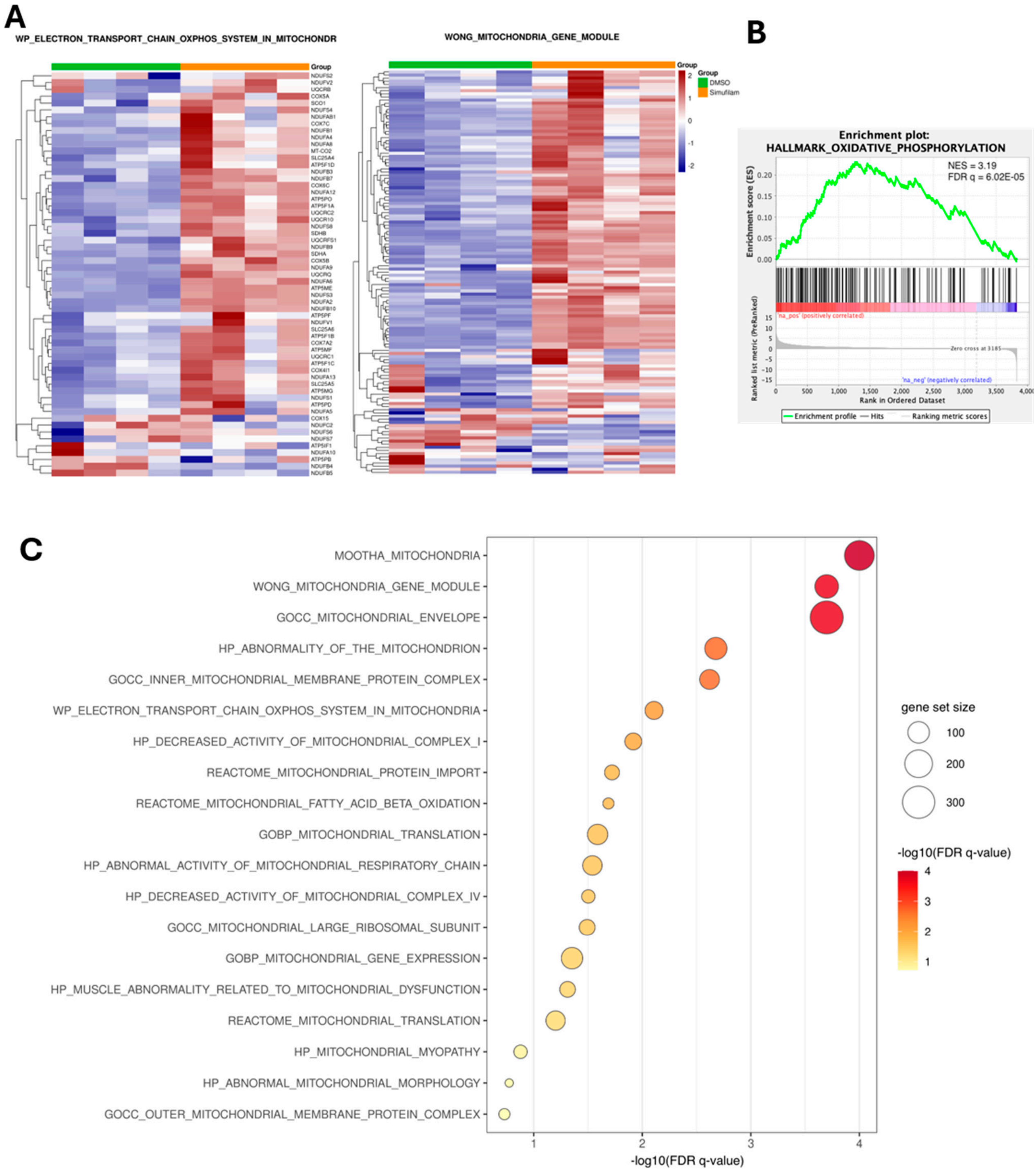

Supplementary Figure S3: GSEA confirms enrichment of mitochondrial and oxidative phosphorylation gene sets following simufilam treatment. (A) Heatmap of leading-edge genes from the WP_electron transport chain_oxphos system_in mitochondria gene set (left) and the

WONG_mitochondria gene module gene set (right) in DMSO- and simufilam-treated *TSC2* KO HEK293T cells. **(B)** GSEA enrichment plot for HALLMARK_oxidative phosphorylation, showing positive enrichment among proteins increased in simufilam-treated cells (NES = 3.19, FDR q = 6.02 x 10^{-5}). The green line indicates the running enrichment score, black vertical bars indicate positions of oxidative phosphorylation genes in the ranked list, and the gray curve indicates the ranked-list metric. **(C)** Bubble plot of significantly enriched mitochondrial-related GSEA signatures. Gene sets were considered enriched at FDR q < 0.25.

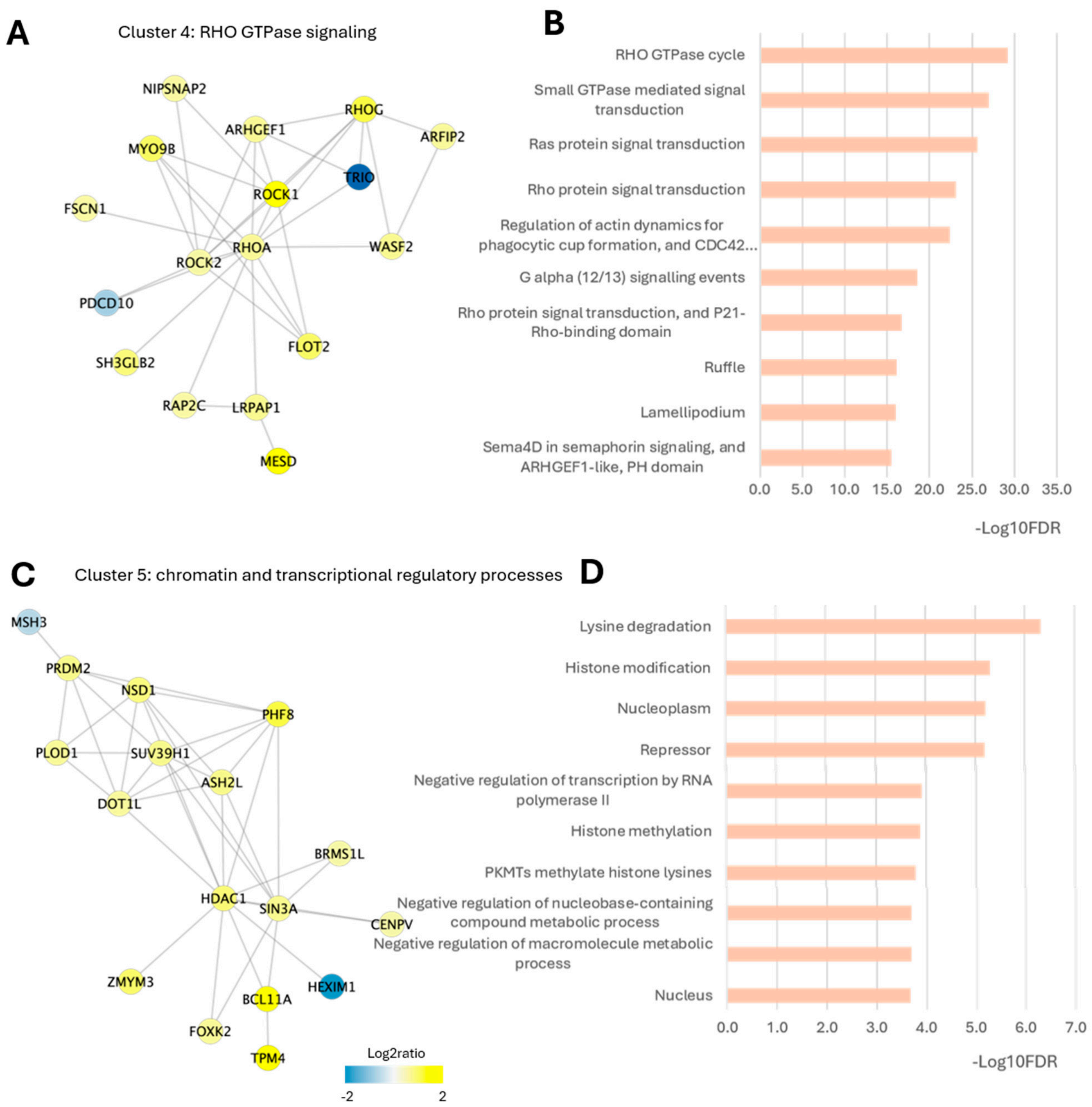

Supplementary Figure S4: Proteomics analysis of simufilam treatment identifies protein clusters enriched in RHO GTPase signaling and chromatin regulatory pathways. (A, C) STRING protein–protein interaction networks of differentially expressed proteins in Cluster 4 and Cluster 5. Node colors indicate log_2ratio fold-change. **(B, D)** Top enriched GO, Reactome, and STRING pathways for each cluster. Enrichment significance was determined using false discovery rate (FDR)-adjusted P values.

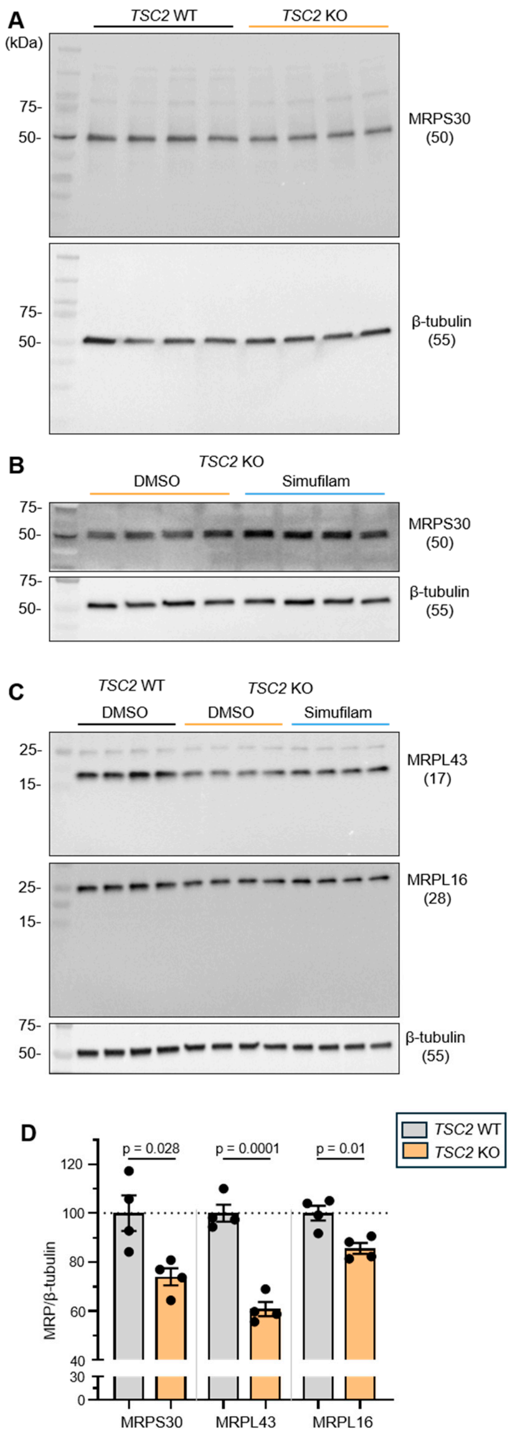

Supplementary Figure S5: *TSC2* deficiency leads to reduced expression of three mitochondrial ribosomal proteins. (A-C) Representative western blot images showing the expression of mitochondrial ribosomal proteins MRPS30 in WT vs KO cells (A), and in KO cells treated with DMSO or simufilam (B), and MRPL43 and MRPL16 in WT cells, and KO cells

treated with DMSO or simufilam (C). **(D)** Quantification of the blots. Data are presented as mean ± SEM. N = 4 per condition as shown on the plots. Statistical analysis: unpaired t-test with Welch's correction. P values are listed on top of each plot.

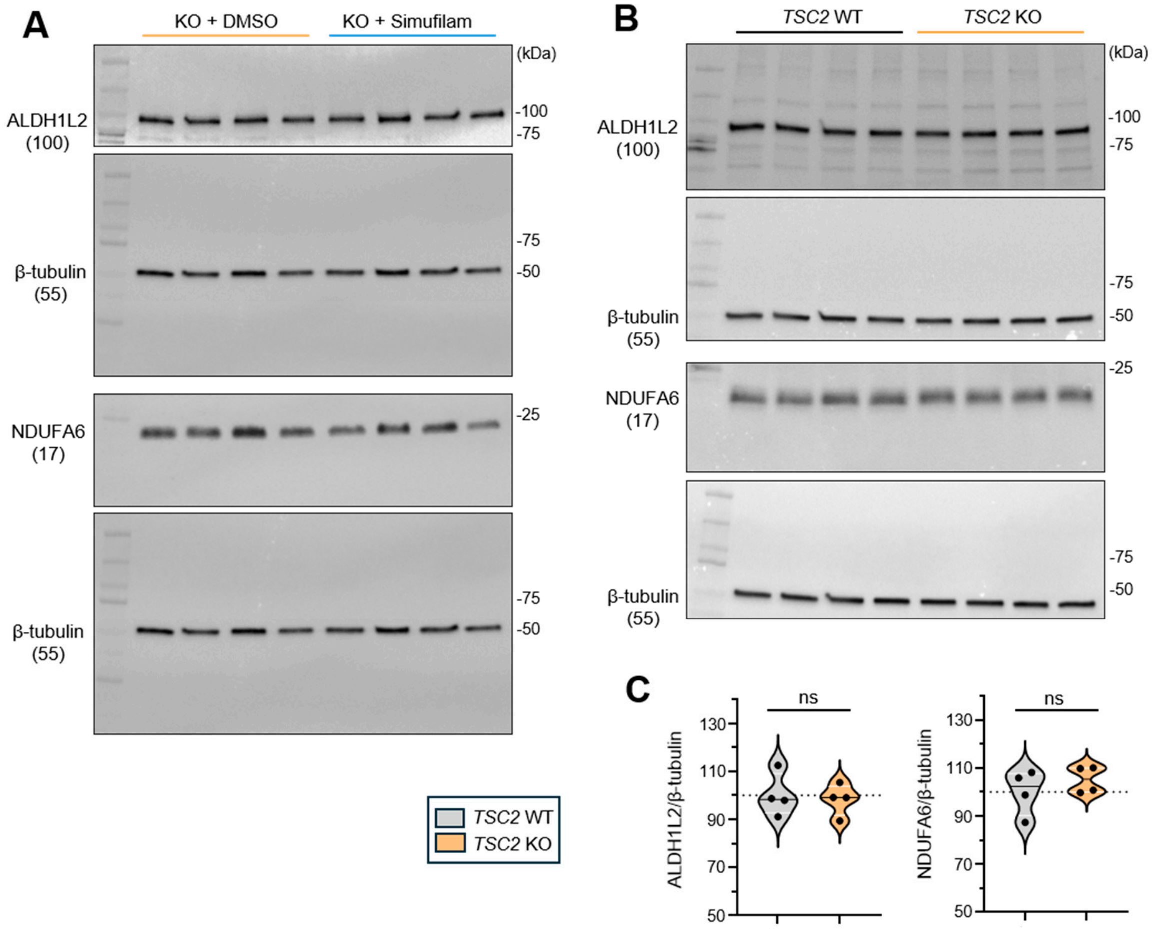

Supplementary Figure S6: (A) Representative western blot images of mitochondrial functional proteins ALDH1L2 and NDUFA6 in *TSC2* KO cells treated with DMSO or simufilam. **(B and C)** Representative western blot images of ALDH1L2 and NDUFA6 in *TSC2* WT and KO (B) and quantification of the blots (C). Data are presented as mean ± SEM. N = 4 per condition as shown on the plots. Statistical analysis: unpaired t-test with Welch's correction. P values are listed on top of each plot.

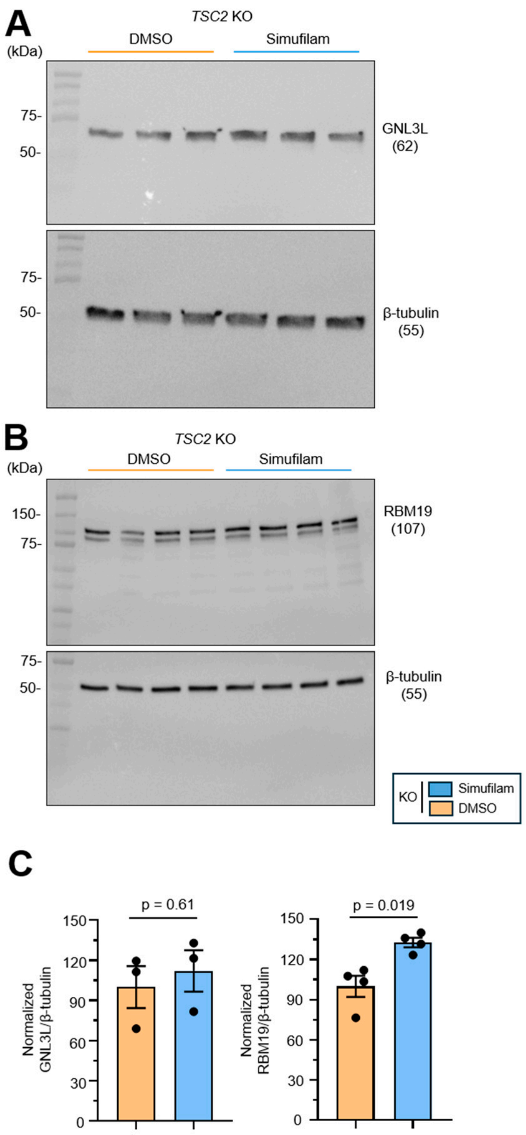

Supplementary Figure S7: Simufilam effect on the two ribosome biogenesis-associated proteins GNL3L and RBM19. (A and B) Immunoblot images of GNL3L (A) and RBM19 (B) in *TSC2* KO cells treated with DMSO or simufilam. **(C)** Quantification of the blots. Data are presented as mean ± SEM. N = 3-4 per condition as shown on the plots. Statistical analysis: unpaired t-test with Welch's correction. P values are listed on top of each plot.

Supplementary Table S1: List of antibodies for immunoblotting

Antibodies	Company name	Catalog number	Dilution
Primary antibodies			
p-S6	Cell Signaling Technology	5364	1:1000
S6	Cell Signaling Technology	2217	1:1000
ATF-4	Cell Signaling Technology	11815	1:1000
ALDH1L2	Proteintech	21391-1-AP	1:1000
NDUFA6	Proteintech	15445-1-AP	1:1000
MRPS30	Proteintech	18441-1-AP	1:1000
MRPL43	Invitrogen	PA5-116773	1:1000
MRPL16	Proteintech	83888-1-RR	1:1000
4E-BP1	Cell Signaling Technology	9644	1:1000
P-4E-BP1	Cell Signaling Technology	2855	1:1000
RBM19	St John's Laboratory Ltd	STJ193714	1:1000
β-tubulin	Cell Signaling Technology	86298	1:2500
GNL3L	Invitrogen	701157	1:1000
Secondary antibodies			
Anti-rabbit	Cell Signaling Technology	7074	1:5000
Anti-mouse	Cell Signaling Technology	7076	1:5000
